# Assessing the microbial diversity and proximate composition of smoked-fermented bushmeat from four different bushmeat samples

**DOI:** 10.5114/bta.2024.135637

**Published:** 2024-03-29

**Authors:** Afia Sakyiwaa Amponsah, Gloria Mathanda Ankar-Brewoo, Herman Erick Lutterodt, Isaac Williams Ofosu

**Affiliations:** 1 Kwame Nkrumah University of Science and Technology, Kumasi, Ghana; 2 Department of Hospitality and Tourism, Sunyani Technical University, Sunyani, Ghana

**Keywords:** microbial diversity, zoonotic pathogens, bushmeat microbiome

## Abstract

The ever-increasing demand for wildlife-derived raw or processed meat commonly known as bushmeat, has been identified as one of the critical factors driving the emergence of infectious diseases. This study focused on examining the bacterial community composition of smoked and fermented bushmeats, specifically grasscutter, rat, rabbit, and mona monkey. The analysis involved exploring 16Sr RNA amplicon sequences isolated from bushmeat using QIIME2. Microbiome profiles and their correlation with proximate components (PLS regression) were computed in STAMP and XLSTAT, respectively. Results indicate the predominance of *Firmicutes* (70.9%), *Actinobacteria* (18.58%), and *Proteobacteria* (9.12%) in bushmeat samples at the phylum level. *Staphylococcus*, *Arthrobacter*, *Macrococcus*, and *Proteus* constituted the core microbiomes in bushmeat samples, ranked in descending order. Notably, significant differences were observed between the bacterial communities of bushmeat obtained from omnivores and herbivores (rat and mona monkey, and grasscutter and mona monkey), as well as those with similar feeding habits (rat and monkey, and grasscutter and rabbit) at the family and genus levels. Each type of bushmeat possessed unique microbial diversity, with some proximate components such as fat in rat samples correlating with *Staphylococcus*, while proteins in Mona monkey correlated with *Arthrobacter* and *Brevibacterium*, respectively. The study underscores public health concerns and highlights probiotic benefits, as bushmeat samples contained both pathogenic and beneficial bacteria. Therefore, future research efforts could focus on improving bushmeat quality.

## Introduction

Meat derived from wild animals is commonly referred to as bushmeat or wildmeat (Nielsen et al., 2014). It serves as a common source of livelihood for numerous impoverished rural dwellers who both consume and trade it. In urban markets, the bushmeat trade is very lucrative due to the ever-increasing demand from urban consumers (Chaves et al., 2017). Urban dwellers are mostly the elite class who are diet-conscious and prefer to eat safe food. Despite its popularity, the increased demand and commercialization of bushmeat in urban areas expose consumers to potential pathogens upon consumption (Paulsen et al., 2016). Nevertheless, there persists a perception of bushmeat as a safer option due to the animals’ nutrition mode and habitat (forest) (Bannor et al., 2022).

The processing of bushmeat primarily involves smoking, drying, and fermentation, with local practices often eschewing sophisticated technology. Instead, a hurdle technology approach is commonly employed, leveraging the impact of various methods such as smoking, drying, fermentation, salting, or curing to enhance the shelf-life and taste of the final product (Paulsen et al., 2016).

Amidst the challenges associated with hunting, processing, storage, and transportation, the consumption of bushmeat raises substantial public health concerns on the continent (Bachand et al., 2012). These concerns primarily stem from the presence of pathogenic and spoilage organisms (Zakpaa et al., 2009), whose activities can impact the safety of the end meat product. Research findings indicate that the supply chain of bushmeat provides a route for transmitting dangerous pathogens and zoonotic diseases to humans, many of which originate from wildlife (Bernstein et al., 2022).

The consumption of bushmeat in Ghana is estimated at 21 410 kg/month, equivalent to about 0.01 kg/person/day, with an annual retail value of approximately U.S. $ 48 000/year, not accounting for seasonal and festive periods and days (Wahab Abdul et al., 2013). Additional reports indicate that in West Africa, bushmeat contributes to 20% of animal protein consumption among rural residents in Nigeria’s rainforest areas, 75% in rural Ghana, and reaches as high as 80 to 90% in Liberia (Kamins et al., 2015). Hunting plays a crucial role, providing between 30 and 80% of the overall protein intake in rural homes in central Africa (East et al., 2005). For example, a study in Tanzania showed that bushmeat consumption ranged from 10.95 to 32.4 kg/capita/year (Brashares et al., 2011).

Despite its importance to the local population, the international market for bushmeat faces challenges due to concerns about disease transmission by microbes originating from consumption. Limited efforts have been made to enhance the quality of bushmeat. However, for a significant portion of the African population, bushmeat remains a delicacy (Chaves et al., 2017). Notably, bushmeat has been linked to emerging infectious diseases such as Ebola, HIV (Murray et al., 2016), SARS, Monkeypox, and Nipah viruses (Temmam et al., 2017). These viruses have hosts belonging to bacterial phyla such as Firmicutes and Proteobacteria (Temmam et al., 2017). This research aims to investigate the bacterial diversity associated with bushmeat sampled from a village near the forest region in the Bono Region of Ghana, aiming to identify zoonotic, infectious, and probiotic signatures that impact consumers’ health.

## Materials and methods

### Materials

Four different types of bushmeat samples – rabbit (*Oryctolagus cuniculus*), bush rat (*Rattus fuscipes*), mona monkey (*Cercopithecus mona*), and grasscutter (*Thryonomys swinderianus*) – were all acquired through gunshot hunting. These samples were purchased from hunters in *Adentia*, a village near the forest zone around Sunyani in the Bono region of Ghana. Over the period from 5^th^ May to 25^th^ July 2021, a total of 42 meat samples were received, distributed as follows: rat (*n* = 12), rabbit (*n* = 10), monkey (*n* = 10), and grasscutter (*n* = 10). The bushmeat was obtained fresh from the hunters upon their return from the forest, where they had spent 24–48 h hunting. Thorough inspection, checking for evidence of gunshot and assessing fur quality to avoid purchasing meat stored for more than 24 h, was conducted.

The weight of each meat sample was measured, fur was removed through smoking, and the meat was individually salted using 100% rock salt, left to rest for 24 h at room temperature, and then subjected to smoking. The smoking process followed a setup described by Essumang (2014), where samples were arranged on a wire mesh platform supported by a circular framework of a perforated metal drum measuring 0.857 m^2^. The fire was allowed to heat up from the base for about 4 h before placing the meat on it for 8 h. The base of the drum, where the fire was made, was stabilized with a pile of sand, and teak tree pieces with an average length of 0.6 m and thickness of 0.05 m were used. An average temperature of 73 was recorded during smoking, monitored using a Monotaro thermometer (CHE-TN430, Japan). The resulting bushmeat products were stored for 28 days in a jute sack at room temperature, and samples were periodically taken for analysis. These samples were placed in sterile plastic bags and immediately transferred into a refrigerated cold box (about 4°C) to the laboratory, where they were stored in freezers (−20°C).

### Molecular analysis (partial 16S rRNA gene analysis of bushmeat samples)

#### DNA extraction, library construction, and sequencing

##### DNA preparation and sequencing

DNA preparation and sequencing were done as previously described by Strejcek et al. (2018). Ten grams of samples underwent homogenization in 90 ml of Ringer’s solution (Oxoid, Basingstoke, U.K.), and a 1 ml aliquot of the resulting homogenate underwent centrifugation at 10 000 × g for 5 min. The pellet containing microorganisms was then collected, and DNA extraction was carried out using the Power Food Microbial DNA Isolation kit (Mo Bio Laboratories, Inc., Carlsbad, CA, USA) according to the manufacturer’s instructions. Quantification of DNA was performed with a Qubit Fluorometer utilizing a Qubit dsDNA B.R. Assay kit (Invitrogen, USA). The quality assessment involved running an aliquot on a 1% agarose gel in TBE buffer. The variable regions V3–V4 of the bacterial 16S rRNA gene were amplified using degenerate PCR primers (Nguyen et al., 2013), specifically 341F (5′−ACTCCTACGGGAGGCA GCAG−3′) and 806R (5′− GGACTACHVGGGTWTCTAAT−3′). Both forward and reverse primers were tagged with Illumina adapter, pad, and linker sequences (Chen et al., 2020). PCR enrichment took place in a 50 μl reaction containing a 30-ng template. The PCR cycling conditions included an initial step at 94 for 3 min, followed by 30 cycles of 94 for 30 s, 56 for 45 s, 72 for 45 s, and a final extension at 72 for 10 min. Purification of PCR products was achieved using AmpureXP beads (Beckman Coulter, 2002) and eluted in the Elution buffer. Library quantification was conducted using the Agilent 2100 bioanalyser (Agilent, USA). Validated libraries were then subjected to sequencing on the Illumina HiSeq platform (BGI, China), adhering to the standard Illumina pipelines and generating two × 300 bp pairedend reads (Liu et al., 2011).

##### Processing of reads

The raw sequencing data underwent a filtration process to generate high-quality clean reads. This involved the removal of primers and adapters using Cutadapt v2.6. Trimmed reads with an average Phred quality value lower than 20 over 30 base pairs (bp) and those whose lengths were reduced to 75% of their original lengths after truncation were excluded. Additionally, reads with ambiguous bases and low-complexity reads were removed. Subsequently, overlapping paired-end reads were merged to create a consensus sequence using FLASH (Fast Length Adjustment of Short reads, v1.2.11) with a minimum overlapping length of 15 bp and a mismatched ratio in the overlapped region of 0.1.

##### Analysis of amplicons

Amplicon sequences were obtained from the portal of the sequencing company, Beijing Genomics Institute, Hong Kong (BGI-HK), and subjected to integrity checks. The analysis of the sequences was conducted using Quantitative Insights into Microbial Ecology 2 (QIIME2 version 2021.4). The raw reads, totaling 624 878, were effectively denoised and merged into nonchimeric amplicon sequence variants (ASVs), resulting in 93 671 ASVs. The Divisive Amplicon Denoising Algorithm (DADA2) was employed for this purpose (Callahan et al., 2016). Taxonomic labels were assigned to the ASVs using the Greengenes2 database-trained NaVve Bayesian Classifier. Subsequently, the feature table (ASV abundance table) and taxonomy table underwent decompression and were exported for further exploration through additional pipelines.

##### Comparative analysis of amplicons

Following QIIME2 processing, the amplicons underwent additional analysis and visualization using Microbiome analysis (Chong et al., 2020). The data were rarefied to the least sample size and normalized using the trimmed mean of *M*-values (TMM) to ensure equal representation of bushmeat samples. Various measures, including Chao1, Shannon, Simpson, and Observed OTUs, were used to determine the alpha diversity of bacterial communities within the bushmeat samples.

For beta diversity analysis, nonmetric multidimensional scaling (NMDS) and principal coordinate analysis (PCoA) were employed, using Bray-Curtis dissimilarity to determine differences in overall diversity estimates among the bushmeat samples. Comparative analysis of microbial communities within the bushmeat samples was conducted using Statistical Analysis of Taxonomic and Functional Profiles (STAMP version 2.1.3) with Fisher’s *t*-test at a 95% confidence interval. Similarity of Percentage Analysis (SIMPER) and linear discriminant analysis (cut-off: LDA > 2.0) were used to identify indicator organisms and predominant bacteria in the bushmeat samples before regression analysis.

To explore the relationship between relevant physiochemical parameters and bacterial communities identified through SIMPER and LefSe analysis, partial least square regression (PLS regression) was applied.

### Proximate composition of bushmeat samples

Analysis of bushmeat samples for moisture, ash, crude fat, and crude protein content followed approved Association of Official Analytical Chemists methods as outlined by Odebunmi et al. (2010). The determinations were collected, and analyses were carried out in triplicates, each performed after the meat had been processed.

## Results and discussion

### Diversity indices and microbial composition among samples

A total of 624 878 high-quality reads from the 16S rRNA were obtained from the investigation of four bushmeat samples. The unprocessed sequence reads per sample were 156 414, 155 640, 155 972, and 156 852 for grasscutter, rat, rabbit, and Mona monkey, respectively, with a mean value of 156 220 reads.

Microbial diversity indices play a crucial role in mathematically describing the microbial community within a sample or making comparisons with other samples (Thukral, 2017). Alpha diversity, in particular, delves into the diversity and richness of species within a functional community, considering both the number of species and the number of individuals of different species in a sample (Thukral, 2017). The Simpson diversity index, a dominance index that places more weight on dominant species, emphasizes relative abundance (Kim et al., 2017).

Table 1 presents the observed number of ASVs for each bushmeat sample, with counts of 73 for grasscutters, 92 for rats, 89 for rabbits, and 61 for mona monkeys. These figures highlight the different numbers of bacterial communities in each bushmeat sample. Notably, the observed number of ASVs is lower than that observed in smoked pig carcasses, where the count reached as high as 1600 (Braley et al., 2022), and dried Hanwoo beef cattle with 855 ASVs (Kim et al., 2020). The number of observed ASVs was highest in rats and lowest in mona monkeys, indicating that both the type of meat and the processing methods play crucial roles in determining the bacterial populations in processed meat (Braley et al., 2022; Han et al., 2014).

**Table 1:** Alpha diversity of bacteria in bushmeat samples

Sample	Quality reads	Observed ASVs	Shannon	Simpson
Grasscutter	156 414	73	2.70	0.87
Rat	155 640	92	1.92	0.70
Rabbit	155 972	89	2.47	0.83
Mona monkey	156 852	61	1.87	0.70

The alpha diversity measures based on the abundance and evenness of bacterial communities in bushmeat indicated that grasscutter meat exhibited the most diverse bacterial community. Conversely, the relatively low value of 1.87 in mona monkey samples implies fewer species and a higher probability of observing specific organisms or the occurrence of dominant bacterial species in mona monkeys. In comparison, previous studies reported a Shannon diversity index ranging from 2.016 to 2.715 in dried aged beef strip lions (Kim et al., 2017), 7.76 in dried Hanwoo beef cattle (Kim et al., 2020), 1.6 in dried buffalo, 1.4 in dried wildebeest, and 1.5 in other types of bushmeat (Katani et al., 2019). Notably, samples classified as bushmeat (wildebeests and buffalo) in Katani et al.’s study exhibited lower Shannon diversity estimates, aligning with the current research results where Shannon diversity values for all samples ranged between 1 and 3. Importantly, all tested bushmeat samples demonstrated diverse bacterial communities irrespective of the type of meat. Interestingly, the higher evenness in bacterial distribution observed in rabbits (0.83) and grasscutters (0.87) compared to other samples may be related to their herbivorous mode of nutrition.

### Taxonomic diversity of bacteria in processed bushmeat samples

**Fig. 1:**
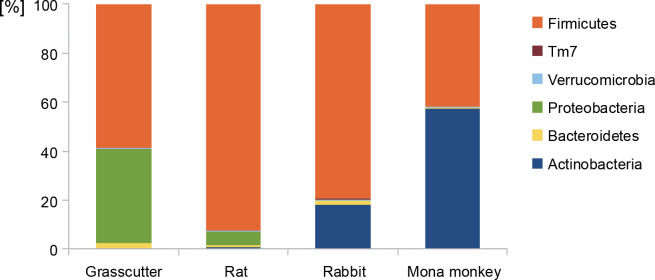
Bar graph showing the most abundant bacterial phyla in grasscutter, rat, rabbit, and mona monkey samples, based on bacterial 16S rRNA gene sequences retrieved from the metagenome analysis; the Y-axis represents the bushmeat samples, and the X-axis represents the percentage abundance of bacterial phyla

**Fig. 2:**
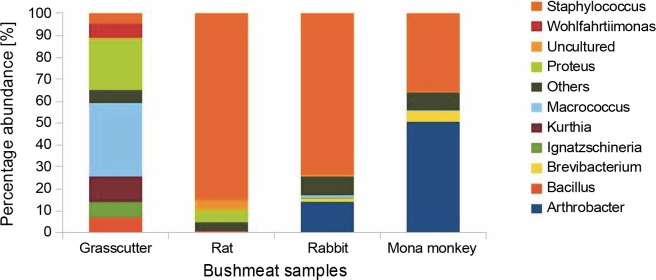
Bar chart showing bushmeat samples' top 10 most abundant bacterial genera; the Y-axis represents the bush meat samples, and the X-axis represents the percentage abundance of bacterial genera

The analysis of bacterial composition in bushmeat samples at the phylum and genus levels is illustrated in Figures 1 and 2. At the phylum level, the grasscutter samples primarily comprised *Firmicutes* (57.4%), *Proteobacteria* (37.5%), and *Bacteroidetes* (0.03%). In rats, the major microbial communities were *Firmicutes* (90.9%), *Proteobacteria* (5.6%), and *Bacteroidetes* (0.02%). Rabbit meat exhibited a core microbiome consisting of *Firmicutes* (79.6%), *Actinobacteria* (18.3%), and *Bacteroidetes* (0.002%), while *Actinobacteria* (57.36%) and *Firmicutes* (41.8%) dominated in mona monkey samples. Overall, the predominant bacterial groups recovered from all bushmeat samples tested included *Firmicutes* (70.9%), *Actinobacteria* (18.58%), *Proteobacteria* (9.12%), *Bacteroidetes* (1.02%), *Verruco-microbia* (0.27%), and TM7 (0.05%) – Figure 1.

Previous studies, such as Lv et al. (2021), have identified *Firmicutes*, *Proteobacteria*, *Cyanobacteria*, *Actinobacteria*, and *Bacteroidetes* in fermented pork, with *Firmicutes* being the dominant phylum across all samples. Similarly, in Tanzanian bushmeat classified as wildebeest (*Connchaetes taurinus*) and buffalo (*Syncerus caffer*), *Firmicutes*, *Proteobacteria*, *Cyanobacteria*, and *Bacteroidetes* constituted the major phyla, with percentages of 67.8, 18.4, 8.9, and 3.1%, respectively (Katani et al., 2019). These findings highlight the prevalence of these phyla in various meat and meat products, as reported by the various authors, though they were present in different amounts. The microbiota of meat and meat products is attributed to the intrinsic features of raw materials and the diverse processing methods employed (Lv et al., 2021).

The presence of *Brevibacteria* in rats (Fig. 2) is of particular interest to the food industry due to their ability to produce amino acids like glutamic acid, a precursor to flavor enhancers such as monosodium glutamate. Additionally, *Brevibacteria* are known to produce essential enzymes like cholesterol oxidase in milk products (Forquin and Weimer, 2014), ectoine in cheese (Buňková et al., 2011), and L-amino acids during the ripening of fermented foods (Forquin and Weimer, 2014). Non-pathogenic species of *Corynebacterium*, also present in rats, find critical applications in various industrial processes, including amino acid and nucleotide production, bioconversion of steroids, hydrocarbon degradation, cheese aging, and enzyme production (Oliveira et al., 2017).

The presence of *Kurthia*, observed in grasscutter samples (Fig. 2), is noteworthy as it is considered an opportunistic pathogen whose formation is highly influenced by environmental conditions (Shaw and Keddie, 1983). *Kurthia gibsonii* has been associated with sexually transmitted zoonotic infections, opportunistic infections, and nongonorrheal urethritis in humans (Kövesdi et al., 2016). This raises concerns about the potential risk of zoonotic transmission associated with the handling and consumption of bushmeat, especially grasscutter meat, which is among the most popular bushmeat sold at market centers. *Staphylococcus saprophyticus* causes uncomplicated urinary tract infections in sexually active females (Chen et al., 2022); thus, its high abundance in rabbit, rat, and mona monkey presents a significant public health concern since women play a vital role in the bushmeat trade in countries such as Ghana, Nigeria, Congo, and Brazil and are highly exposed to bushmeat (Babatunde et al., 2020; Chaves et al., 2017; Dery et al., 2022; Kamins et al., 2015).

Predicting whether bacterial species found in bushmeat are inherently present in the host or acquired during the capture, butchering, and transportation process is complex. Nonetheless, the presence of human pathogens in bushmeat can be attributed to inadequate hygienic practices during hunting or meat processing, as hunters and butchers may not adhere to suitable hygiene protocols (Babalola and Oladipupo Azeez, 2018; Hilde-rink and de Winter, 2021; Rakotoarivony et al., 2022). The human skin microbiota primarily comprises *Proteobacteria*, *Firmicutes*, and *Bacteroidetes* (Ashaolu et al., 2021). Poor hygiene during handling and processing could lead to cross-contamination from human skin to meat, potentially exposing meat consumers to these microorganisms.

Several precapture factors may influence the microbiota communities present in bushmeat. These include the movement of wildlife through different habitats and prevailing environmental conditions, which can increase disease susceptibility (Barboza et al., 2016; Glover, 2014; Van Vliet et al., 2017; Wiafe, 2014). Other factors include the feeding habits of different wildlife species (grazer or browser, height at which they feed on vegetation), the social organization of the species (e.g., solitary or gregarious), and the interactions of wildlife with other domestic and wild species (Paulsen et al., 2016).

The data in Table 2 shows that while some bacterial genera are common across bushmeat samples, certain genera are unique to specific bushmeat samples: grasscutter – *Myroides*, *Anaerosinus*, *Flexispira*, *Serpens*, *Gaiella*, *Peptostreptococcus*, rat – *Paraprevotella*, *Rhodanobacter*, *Terribacillus*, *Burkholderia*, *Pedobacter*, *Massilia*, *Butyricicoccus*, *Desulfovibrio*, rabbit – *Sphingomonas*, *Oceanobacillus*, *Selenomonas*, *Nocardiopsis*, and monkey – *Sporobacter*, *Ornithinibacillus*, *Subdoligranulum*.

**Table 2:** Co-occurrence of bacterial genera communities and unique signatures of each in Bushmeat sample

Samples	Co-occurrences	Bacteria Genera
Monkey/ Grasscutter/ Rabbit/ Rat	17	*Bifidobacterium, Prevotella, Arthrobacter, Bacteroides, Micrococcus, Clostridium, Succinivibrio, Ruminococcus, Akkermansia, Faecalibacterium, Dialister, Ignatzschineria, Brevibacterium, Roseburia, Staphylococcus, Succinispira, Proteus*
Grasscutter/ Rabbit/ Rat	2	*Kurthia, Providencia*
Monkey/Rabbit/Rat	1	*Corynebacterium*
Grasscutter/ Rat	5	*Macellibacteroides, Anaerorhabdus, Bacillus, Collinsella, Lactobacillus*
Monkey/Grasscutter	2	*Weissella, Wohlfahrtiimonas*
Rabbit /Rat	3	*Streptococcus, Dorea, Alistipes*
Monkey/ Rat	1	*Gemmiger*
Grasscutter	6	*Myroides, Anaerosinus, Flexispira, Serpens, Gaiella, Peptostreptococcus*
Rat	8	*Paraprevotella, Rhodanobacter, Terribacillus, Burkholderia, Pedobacter, Massilia, Butyricicoccus, Desulfovibrio*
Rabbit	4	*Sphingomonas,Oceanobacillus, Selenomonas, Nocardiopsis*
Monkey	3	*Sporobacter,Ornithinibacillus, Subdoligranulum*

**Fig. 3:**
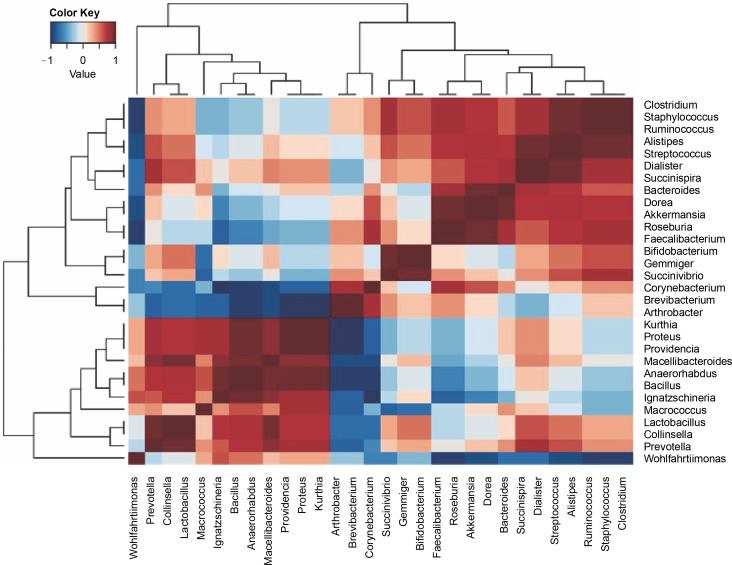
A heat map of the bacterial genera was obtained from all the bushmeat samples, showing the dominant genera’s positive (red) and negative (blue) correlation using Ward's clustering method

All the samples exhibited 17 dominant bacterial genera, as detailed in Table 2. These genera included *Bifidobacterium, Prevotella, Arthrobacter, Bacteroides, Macrococcus, Clostridium, Succinivibrio, Ruminococcus, Akkermansia, Faecalibacterium, Dialister, Ignatzschineria, Brevibacterium, Roseburia, Staphylococcus, Succinispira*, and *Proteus*. The heatmap in Figure 3 illustrates positive correlations among *Bifidobacterium, Succinivibrio, Ruminococcus, Akkermansia, Feacalibacteria, Roseburia, Staphylococcus*, and *Succinispira*. These bacterial groups were identified as core components in all samples, indicating their potential role in meat processing. Additionally, *Wohlfahrtiimonas, Collinsella, Lactobacillus, Micrococcus, Ignatzschinerta, Providentia, Proteus*, and *Kurtia* also exhibited positive correlations in the lower left quadrant.

Previous reports have highlighted *Macrococcus* and *Staphylococcus* as major genera found in pressed salted ducks in China (Wang et al., 2021), and *Clostridium* and *Staphylococcus* as the most dominant in bushmeat samples in Tanzania (Katani et al., 2019). These variations were attributed to differences in raw materials, technology, auxiliary materials, and fermentation time (Wang et al., 2021). The presence of lactic acid bacteria (*Lactobacillus* ) suggests the low spoilage potential of smoked bushmeat (Table 3).

**Table 3:** Proximate composition of smoked bushmeat

Bushmeat	Moisture [%]	Total ash [%]	Crude fat [%]	Crude fiber [%]	Crude protein	NFE
Rabbit	18.73	7.78	8.79	0.11	20.08	44.69
Rat	18.67	7.78	9.23	0.11	21.35	42.79
Grasscutter	15.94	2.06	6.27	0.27	26.29	49.29
Mona monkey	17.97	2.34	7.12	0.22	29.80	45.62

While the presence of *Clostridium* and *Staphylococcus* could affect the health of individuals consuming bushmeat, as these organisms have been reported as pathogenic (Bartkiene et al., 2017) and are often implicated in food poisoning cases (Magnino et al., 2009), the co-occurrence of probiotic strains such as *Bifidobacterium*, *Bacteroides*, and *Succinospira* may contribute to both the taste and probiotic benefits for consumers, aiding in the maintenance of gut bacterial flora.

### Pairwise comparison of microbiota among two bushmeat samples

**Fig. 4:**
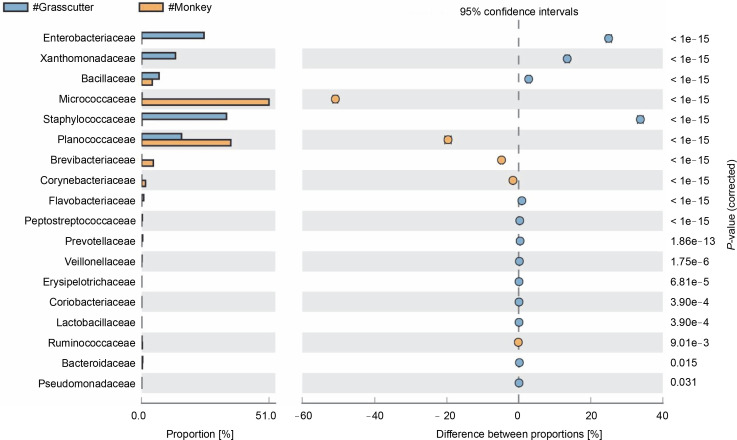
Comparative analysis of bacteria families in grasscutter (herbivore) and mona monkey (omnivore) meat based on their mode of nutrition (*P* < 0.05; Fisher's exact *T*-test)

**Fig. 5:**
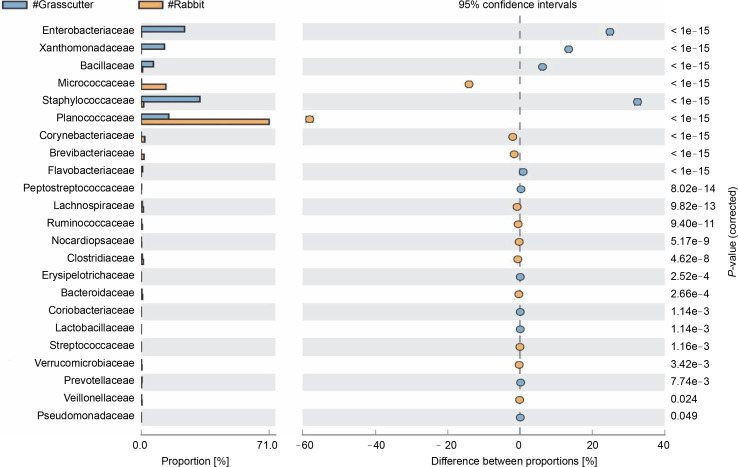
Comparative analysis of bacteria families in grasscutter (herbivore) and rabbit (herbivore) meat based on their mode of nutrition (*P* < 0.05; Fisher's exact *T*-test)

The comparison of microbiota in assessed bushmeat samples from animals with the same feeding habits (herbivore or omnivore) and those with different feeding habits revealed that the animals' feeding habits did not significantly influence the abundance of bacteria, as shown in Figures 4 and 5, as well as in Supplementary Figures 1–3. Nevertheless, in mona monkey and grasscutter samples, there was no significant difference (*P* < 0.05) in *Pseudomonadaceae* proportions (Fig. 4). It was noted that the proportions of *Micrococcaceae*, *Enterobacteriaceae*, *Staphylococcaceae*, and *Planococcaceae* were consistently high across all samples (Fig. 4, Fig. 5 and Suppl. Fig. 1–3).

The presence of *Macrococcus brunensis*, *Succinispira mobilis*, and *Akkermansia muciniphila*, considered next-generation probiotics due to their role in treating metabolic disorders associated with obesity and type 2 diabetes (Cani and de Vos, 2017), along with *Lactobacillus paraplantarum* used as an adjunct culture to enhance cheese quality (Boukria et al., 2020), and *Bifidobacterium* breve (Forquin and Weimer, 2014), especially in grasscutter meat, supports the rife perception of some beneficial impacts of consuming bushmeat.

While the diet of animals can influence the microbial community in their digestive tract, such as herbivores harboring different microbes than carnivores or omnivores (Porto et al., 2017), this study suggests that each bushmeat sample is unique in its microbial abundance, even among samples with the same feeding group.

### Potential correlation of microbiota, pH, and proximate composition of bushmeat

Microbial diversity and proximate composition, which encompass the physicochemical properties of food products, are crucial factors in understanding the quality and safety of these products (Osimani et al., 2017).

Proximate composition refers to the chemical makeup of a food product, including levels of fat, protein, nitrogen-free extract (NFE), fiber, moisture, and ash (Akonor et al., 2016). Grasscutters exhibited high protein content (26.29%), surpassing the reported protein content of grasscutters (21.12%) by Wogar et al. (2012). The fat content of rats (8.79%) falls below the 15% reported for roasted cane rats by Emelue and Ikoyo (2017). In a prior study, the crude protein of rabbits was noted as 14.25% (Wogar et al., 2012), which is lower than the percentage reported in this study. Variations in proximate composition among animals can be attributed to factors such as processing methods, feeding nature (controlled or wild feeding), and the health of the animal (Emelue and Ikoyo, 2017).

**Fig. 6:**
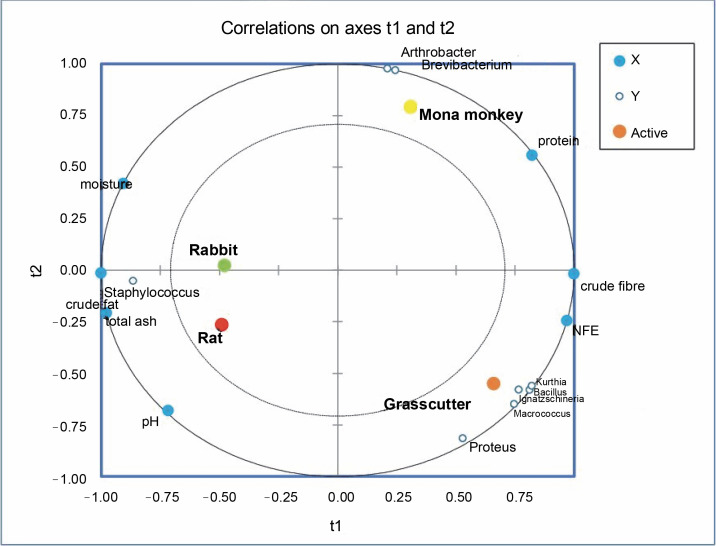
Partial least square regression correlation plot of bacterial communities and physicochemical parameters in bushmeat

Partial least square regression showed strong correlations between physicochemical parameters like moisture, total ash, pH, and crude fat with the abundance of *Staphylococcus* in rabbit and rat meat (Fig. 6). Crude fiber and nitrogen-free extract (NFE) levels exhibited strong correlations with the abundance of *Bacillus*, *Ignatzschineria*, *Kurthia*, *Proteus*, and *Macrococcus* in the grasscutter. On the other hand, crude protein levels were associated with *Arthrobacter* and *Brevibacterium* in Mona monkeys. All physicochemical parameters were identified as relevant factors influencing the distribution of bacterial communities in bushmeat (Suppl. Table 1).

Recent studies have noted a correlation between microbial diversity and the proximate composition of various food products, such as dried fishery products (Rasul et al., 2022), edible insects (Osimani et al., 2017), and dairy products (Boukria et al., 2020).

The PLS regression results reveal that the abundance of *Staphylococcus saprophyticus* is associated with the acid content, moisture, total ash, and crude fat levels in rat and rabbit meat. A similar observation in smoked Suya in Nigeria, with high moisture content (39.65%), suggests a high risk of deterioration and the presence of food-borne pathogens like *Escherichia coli*, *Staphylococcus aureus*, and *Salmonella typhi* (Adeyeye et al., 2022). Proximate analysis results indicate that the highest protein levels (29.80%) observed in mona monkey meat correlate with the abundance of *Brevibacterium casei* and *Arthrobacter castelli*. While the latter has not been associated with infections or food contamination, *B. casei* has been linked to conditions such as brain abscesses (Kandasamy et al., 2018), sepsis in immunocompromised individuals (Todorov et al., 2017) and catheter-related bloodstream infections (Kövesdi et al., 2016), thus suggesting a potential infectious concerns in the consumption of mona monkey meat. Despite the predominance of *S. saprophyticus* in rat meat, the presence of bacteria like *Bifidobacterium breve*, *Clostridium bolteae*, *Bacteroides acidifaciens*, *Succinispira mobilis*, *Akkermansia muciniphilia*, and *Macrococcus brunensis* indicates the probiotic potential of this ruminant meat.

Certain microorganisms thrive in high-fat environments, as observed in rats where *Staphylococcus* correlates well with crude fat. *Staphylococcus* spp. are known to break down branched fatty acids in the host organism’s skin (Jackson, 2006). In contrast, bacterial species like *Macrococcus* and *Proteus* may prefer highcarbohydrate environments (Villasante et al., 2019), as seen in the grasscutter. Thus, the proximate composition of a food product can influence the diversity and abundance of microorganisms present in it.

Overall, understanding the relationship between microbial diversity and proximate composition is essential for ensuring food safety and quality. Monitoring these factors allows food manufacturers to exercise better control over the production process, ensuring that products are both safe and nutritious (Akonor et al., 2016).

The intake of bushmeat carries a possible human health risk. The bushmeat samples contained higher proportions of *Firmicutes* and *Proteobacteria*, which can host most zoonotic viruses, therefore suggesting the need for further studies of bushmeat as a possible worldwide health problem.

## Conclusion

Bushmeat samples exhibit unique and diverse microbial communities, regardless of their feeding habits. Molecular signatures of potentially dangerous zoonotic pathogens were identified, including organisms from the families *Planococcaceae*, *Staphylococcaceae*, *Micrococcaceae*, *Xanthamonadaceae*, and *Veillonellaceae*. The work also reports the correlation between proximate composition and microbial diversity, suggesting that specific composition directly impacts the microbial makeup of meat. Various factors such as animal diet, environmental factors, animal health, hunting practices, processing methods, and human contamination play roles in shaping the bushmeat microbiome. This study also suggests the need for future investigation to assess potential health risks associated with bushmeat hunting, trade, and consumption.

## Declaration of interest

The authors declare no conflict of interest.

## Supplementary Material

Assessing the microbial diversity and proximate composition of smoked-fermented bushmeat from four different bushmeat samples
