# Supplementary material for: Assessing the microbial diversity and proximate composition of smoked-fermented bushmeat from four different bushmeat samples
Source: BioTechnologia (Pozn). 2024 Mar 29;105(1):5–17. doi: 10.5114/bta.2024.135637 (PMC11020155; doi:10.5114/bta.2024.135637)
Supplement: Assessing the microbial diversity and proximate composition of smoked-fermented bushmeat from four different bushmeat samples [file BTA-105-1-52453-s001.pdf]

BioTechnologia vol. 105 (1) • 2024
Journal of Biotechnology, Computational Biology and Bionanotechnology SUPPLEMENTARY MATERIALS
https://doi.org/10.5114/bta.2024.135637

CC BY 4.0

# Assessing the microbial diversity and proximate composition of smoked-fermented bushmeat from four different bushmeat samples

AFIA SAKYIWAA AMPONSAH *, GLORIA MATHANDA ANKAR-BREWOO, HERMAN ERICK LUTTERODT, ISAAC WILLIAMS OFOSU
Kwame Nkrumah University of Science and Technology, Kumasi, Ghana

Received: 9 December 2023; revised: 5 January 2024; accepted: 5 January 2024

### Abstract

The ever-increasing demand for wildlife-derived raw or processed meat commonly known as bushmeat, has been identified as one of the critical factors driving the emergence of infectious diseases. This study focused on examining the bacterial community composition of smoked and fermented bushmeats, specifically grasscutter, rat, rabbit, and mona monkey. The analysis involved exploring 16Sr RNA amplicon sequences isolated from bushmeat using QIIME2. Microbiome profiles and their correlation with proximate components (PLS regression) were computed in STAMP and XLSTAT, respectively. Results indicate the predominance of *Firmicutes* (70.9%), *Actinobacteria* (18.58%), and *Proteobacteria* (9.12%) in bushmeat samples at the phylum level. *Staphylococcus*, *Arthrobacter*, *Macrococcus*, and *Proteus* constituted the core microbiomes in bushmeat samples, ranked in descending order. Notably, significant differences were observed between the bacterial communities of bushmeat obtained from omnivores and herbivores (rat and mona monkey, and grasscutter and mona monkey), as well as those with similar feeding habits (rat and monkey, and grasscutter and rabbit) at the family and genus levels. Each type of bushmeat possessed unique microbial diversity, with some proximate components such as fat in rat samples correlating with *Staphylococcus*, while proteins in Mona monkey correlated with *Arthrobacter* and *Brevibacterium*, respectively. The study underscores public health concerns and highlights probiotic benefits, as bushmeat samples contained both pathogenic and beneficial bacteria. Therefore, future research efforts could focus on improving bushmeat quality.

**Key words:** microbial diversity, zoonotic pathogens, bushmeat microbiome

Supplementary Table 1. Variables of importance for physicochemical (VIP) parameters in smoked bushmeat

| Variable | VIP(1) | Lower bound (95%) | Upper bound (95%) | VIP(2) | Lower bound (95%) | Upper bound (95%) | VIP(3) | Lower bound (95%) | Upper bound (95%) |
|---|---|---|---|---|---|---|---|---|---|
| pH | 0.454 | −2.245 | 3.153 | 1.301 | 0.090 | 2.512 | 1.341 | 0.217 | 2.464 |
| Moisture | 1.257 | −0.951 | 3.464 | 1.170 | 0.327 | 2.014 | 1.151 | 0.313 | 1.989 |
| Protein | 0.577 | −1.508 | 2.663 | 1.118 | 0.111 | 2.126 | 1.144 | 0.223 | 2.065 |
| NFE | 1.216 | −0.272 | 2.705 | 0.955 | −0.004 | 1.915 | 0.930 | −0.032 | 1.892 |
| Total ash | 0.960 | 0.425 | 1.496 | 0.769 | 0.390 | 1.148 | 0.764 | 0.410 | 1.117 |
| Crude fibre | 1.123 | 0.598 | 1.648 | 0.776 | −0.031 | 1.583 | 0.755 | −0.037 | 1.547 |
| Crude fat | 1.104 | 0.706 | 1.501 | 0.762 | 0.015 | 1.509 | 0.742 | 0.013 | 1.471 |

* Corresponding author: Kwame Nkrumah University of Science and Technology, Kumasi, Ghana; e-mail: afia.amponsah@stu.edu.gh

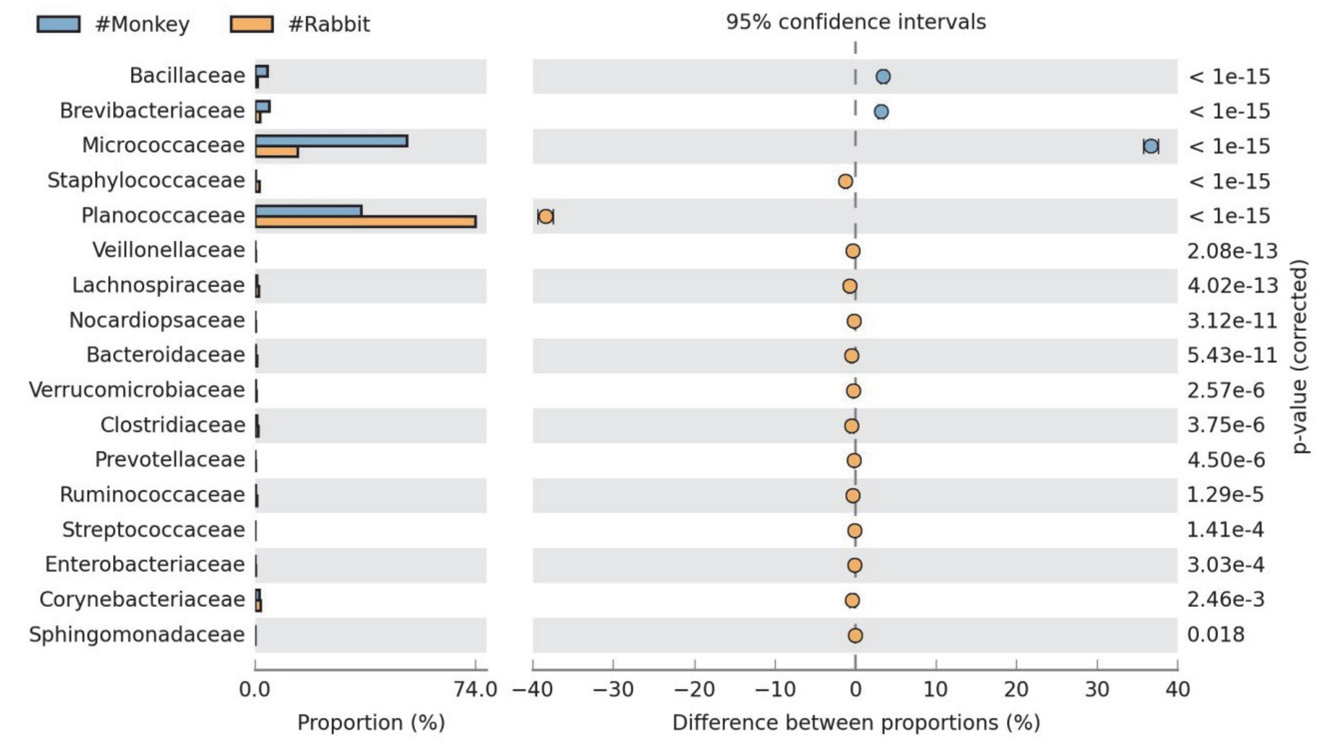


Supplementary Fig. 1. Comparative analysis of bacteria families in mona monkey (omnivore) and rabbit (herbivore) meat based on their mode of nutrition ($P < 0.05$; Fisher's exact T-test)

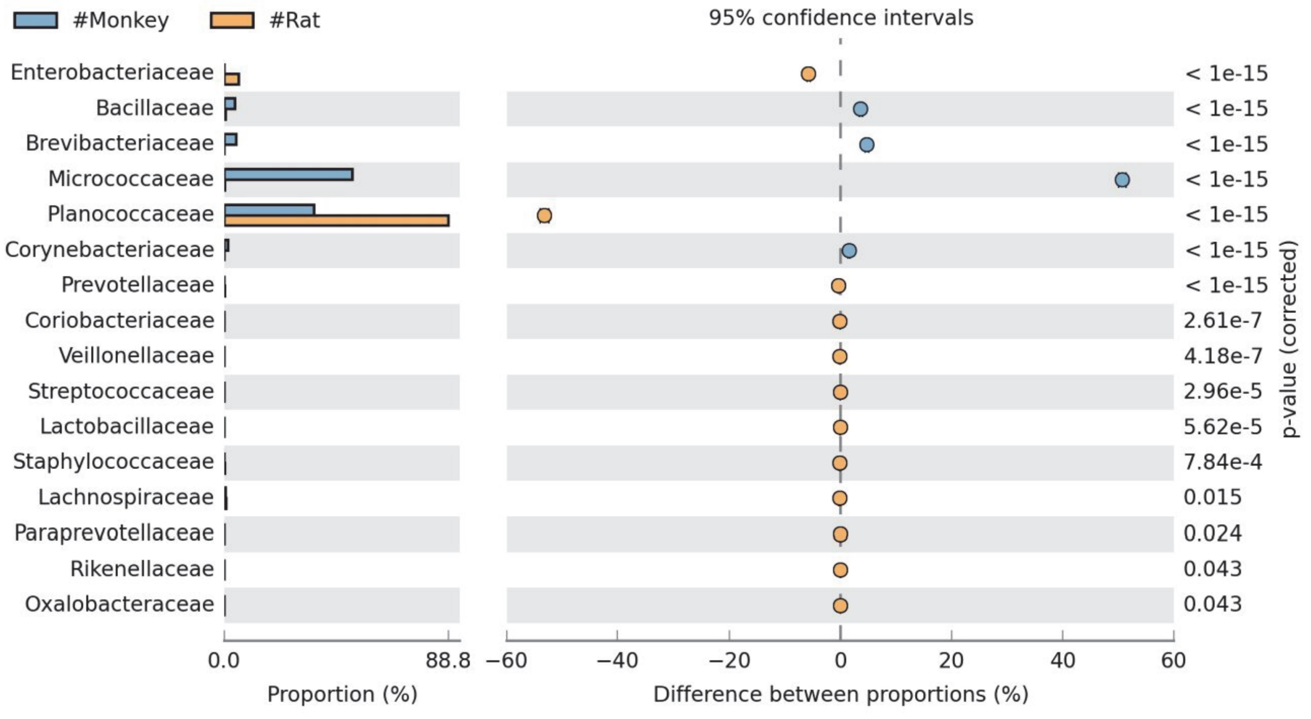


Supplementary Fig. 2. Comparative analysis of bacteria families in smoked mona monkey (omnivore) and rat (omnivore) meat based on their mode of nutrition ($P < 0.05$; Fisher's exact T-test)

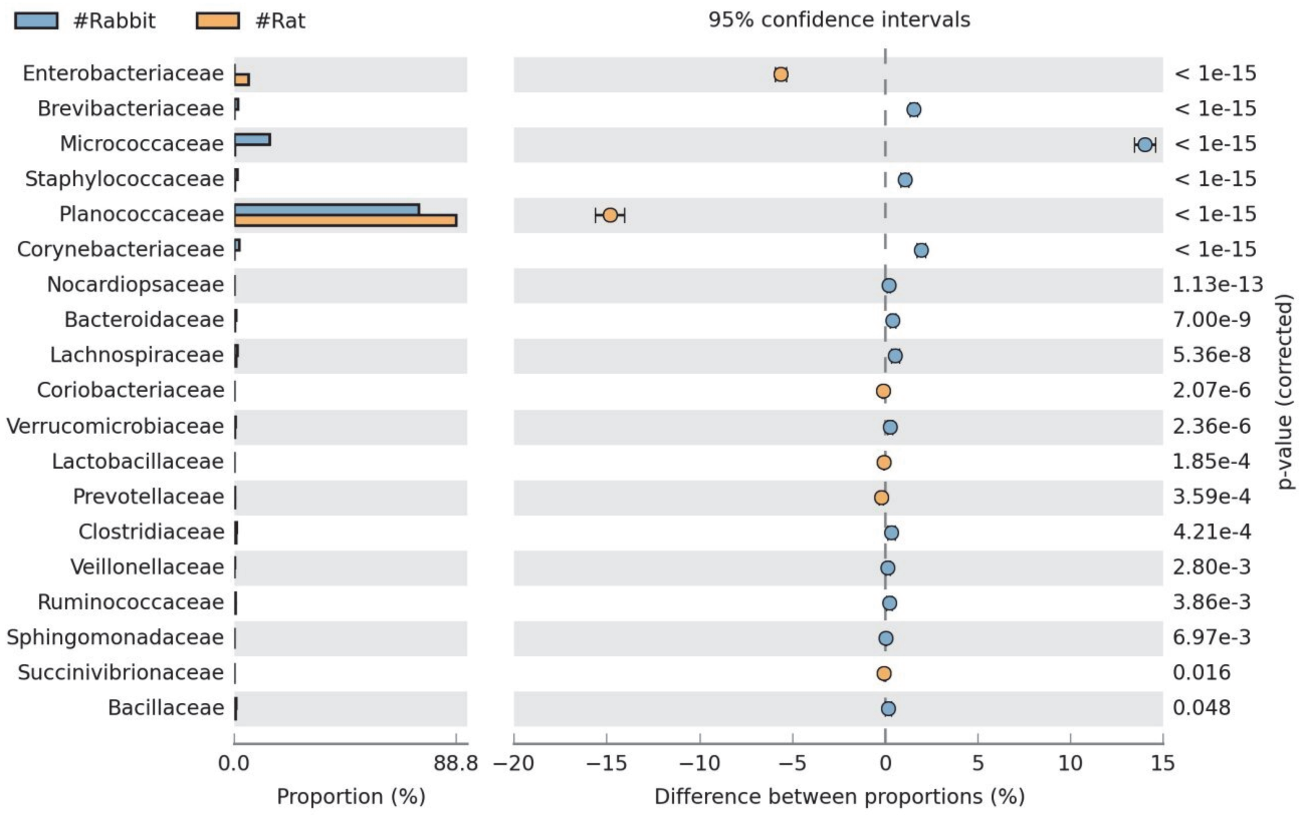


Supplementary Fig. 3. Comparative analysis of bacteria families in rabbit (herbivore) and rat (omnivore) meat based on their mode of nutrition ($P < 0.05$; Fisher's exact T-test)
